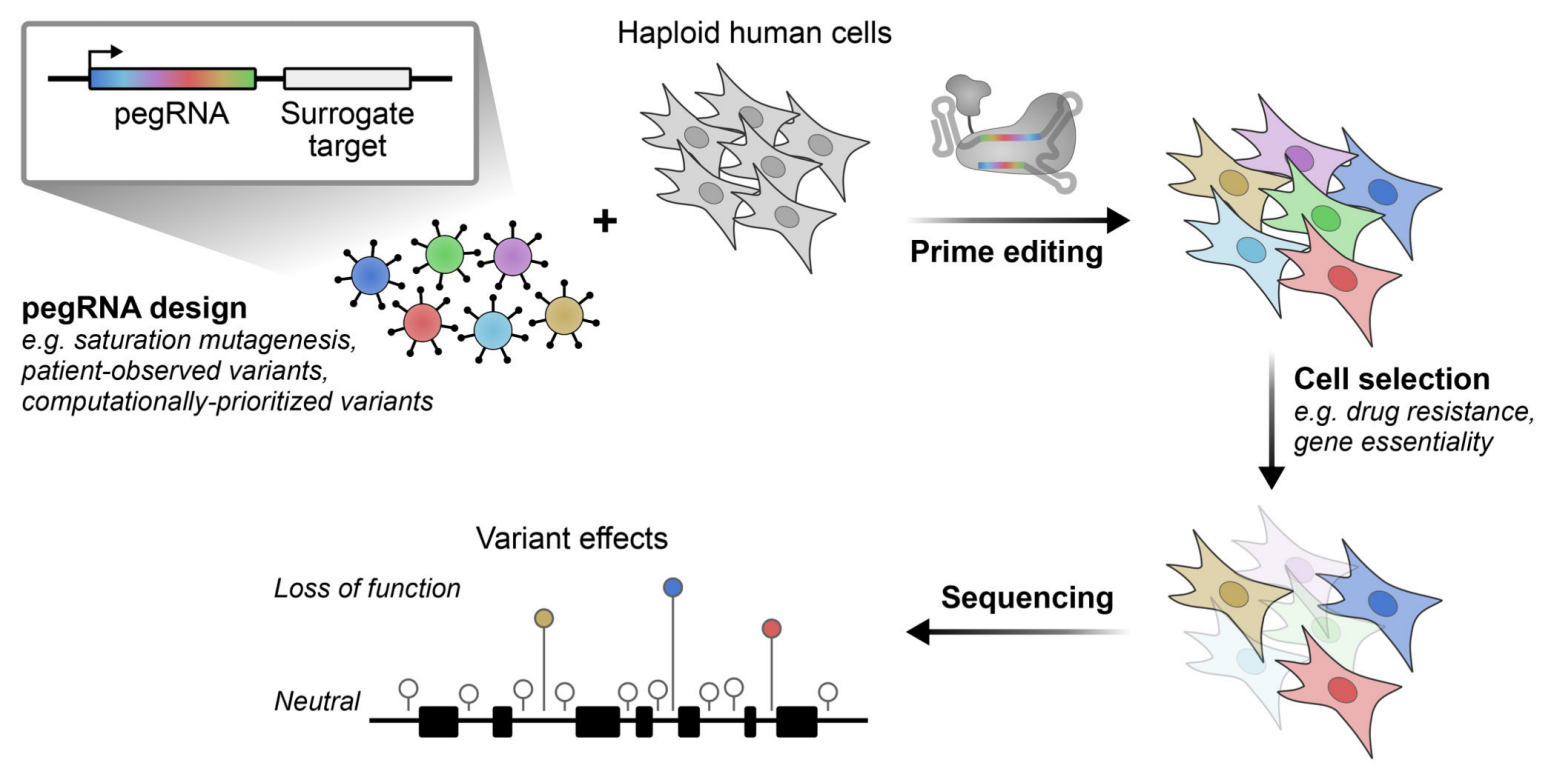# Determining variant effects with pooled prime editing

**DOI:** 10.1038/s41576-025-00865-8

**Published:** 2025-10-01

**Authors:** Christina M. Kajba, Michael Herger

**Affiliations:** 1 The Genome Function Laboratory, The Francis Crick, Institute, London, UK

Over one billion genetic variants have been identified in humans. Yet, most of these variants lack functional characterization and it remains unclear whether they impact human health. Multiplexed assays of variant effect (MAVEs) can close this gap by enabling the phenotypic consequences of thousands of genetic variants to be determined in a single experiment. Among MAVEs, assays that use genome editing offer a unique advantage: they test variants in their native context, thereby capturing effects on both gene expression and protein function. Still, editing-based MAVEs are limited regarding the number and type of variants that can be tested.

First described by Anzalone et al, prime editing stands out as a potentially powerful solution because it allows virtually any short variant anywhere in the human genome to be installed with high precision. Prime editing relies on two main components: the prime editor and the prime editing guide RNA (pegRNA). The prime editor, a fusion of a Cas9 nickase with a reverse transcriptase, writes the desired edit into the genome. The pegRNA both specifies the genomic target site and provides the template for the genetic change. Low editing efficiencies have hampered efforts to use prime editing for variant screening, but recent improvements to the technology have made such screens possible.

**Commented [DC1]:** We don’t use citation numbers in tools of the Trade articles.

In our study, we built upon prime editing advances to develop a new screening platform that enables the functional characterization of variants at scale (Fig. 1). First, variants of interest are defined and a corresponding pegRNA library is designed and cloned. This library is then introduced via lentiviral delivery to a population of cells expressing the prime editor. We use HAP1 cells in our platform, as their haploid nature allows variant effects to manifest without the influence of a second allele. Once editing has occurred, cells are selected based on a relevant phenotype, for example, viability when targeting an essential gene or assaying drug resistance. Causative variants are identified by sequencing the genomically integrated pegRNA constructs before and after selection.

**Commented [DC2]:** Please provide a short caption for Figure 1.

**Commented [MH3R2]:** Caption: Workflow for pooled prime editing screening, enabling the functional characterization of diverse variants in their native context across large genomic regions.

To achieve higher editing efficiencies, we implemented several established prime editing optimizations, including an improved prime editor (PEmax), the use of engineered pegRNAs resistant to degradation, and manipulation of the cells’ DNA repair pathways to favour successful editing. We also incorporated a ‘surrogate target’ to report on the activity of each pegRNA throughout the screen. This is a short copy of the pegRNA’s genomic target that is delivered with each pegRNA and later sequenced, allowing low-activity pegRNAs to be removed during analysis to improve data quality.

We demonstrated our prime editing platform by screening ~930 variants across two tumour suppressor genes – *SMARCB1* and *MLH1*. In *SMARCB1*, we programmed nearly all possible single nucleotide changes within two exons. As *SMARCB1* is essential in HAP1 cells, efficiently introduced loss-of-function variants lead to cell death. In *MLH1*, we showcased the platform’s ability to interrogate extensive genomic regions. We screened ~360 clinically reported non-coding variants distributed across the full 60-kb gene. The readout here was resistance to 6-thioguanine, conferred by loss of MLH1 function in mismatch repair. Our screens and subsequent validation experiments revealed previously unknown deleterious variants, both in coding and non-coding regions.

Together with several other recently described platforms for prime editing screening, our work highlights the potential of prime editing to inform on variant effects at scale. With more than 2,000 genes being essential in HAP1, our platform presents a path forward for identifying loss-of-function variants via gene essentiality. Moreover, our method is uniquely suited for performing large-scale screens of non-coding variants, for which preservation of genomic context is key.

Looking ahead, we anticipate that pooled prime editing screens will play an important role in identifying many new variants underlying human disease.

## Figures and Tables

**Fig 1:**